# Different Antimicrobial Susceptibility Testing Methods to Determine Vancomycin Susceptibility and MIC for *Staphylococcus aureus* with Reduced Vancomycin Susceptibility

**DOI:** 10.3390/diagnostics12040845

**Published:** 2022-03-29

**Authors:** Hae-Sun Chung, Miae Lee

**Affiliations:** 1 Department of Laboratory Medicine, Ewha Womans University College of Medicine, Seoul 07804, Korea; miae@ewha.ac.kr; 2 Ewha Education and Research Center for Infection, Seoul 07985, Korea

**Keywords:** *Staphylococcus aureus*, vancomycin, antimicrobial susceptibility test

## Abstract

The methods and results obtained using commercialized automation systems used for antimicrobial susceptibility testing are not entirely consistent. Therefore, we evaluated different antimicrobial susceptibility testing methods to determine vancomycin susceptibility and minimum inhibitory concentration (MIC) for *Staphylococcus aureus* with reduced vancomycin susceptibility (SA-RVS). A total of 128 clinical isolates of *S. aureus* were tested, including 99 isolates showing an MIC of ≥2 µg/mL using the VITEK2 system (VITEK2). Antimicrobial susceptibility tests were performed using the Sensititre system (Sensititre), Phoenix M50 system (Phoenix), and MicroScan WalkAway 96 Plus system (MicroScan). Vancomycin MICs were determined using the broth microdilution method (BMD) and Etest. Essential agreement and category agreement for each method were compared with BMD results as the reference method. The BMD and Etest showed complete essential agreement (100%). VITEK2, Sensititre, and Phoenix showed high essential agreement (>99%), while MicroScan showed the lowest essential agreement (92.2%). The MIC MICs determined via Etest, VITEK2, and MicroScan tended to be higher than that determined via BMD. When comparing BMD with Etest, the category agreement was 93.8% and minor errors were observed for eight isolates. VITEK2, Sensititre, and Phoenix showed category agreements of 96.1%, 96.1%, and 99.2%, respectively, while MicroScan showed the lowest category agreement of 85.2%. The determination of vancomycin susceptibility and MIC for *S. aureus* varied among the methods. Caution should be taken when interpreting RVS and intermediate results for *S. aureus*. For confirmation of SA-RVS results, it would be appropriate to test with BMD or a more reliable testing method.

## 1. Introduction

*Staphylococcus aureus* is one of the most frequently isolated bacteria in clinical specimens and is considered as an important pathogen that causes infectious diseases in various parts of the body, including skin and soft tissue infections, osteoarthritis, bacteremia, pneumonia, and food poisoning, among others [1]. With the emergence of penicillin-resistant strains, methicillin was introduced in 1960 to treat *S. aureus* infections; however, methicillin-resistant *S. aureus* (MRSA) was subsequently isolated in 1961, and the number of antibiotic-resistant bacteria has continued to increase thereafter [2]. Furthermore, strains showing reduced susceptibility to vancomycin, the only known therapeutic agent for MRSA infections, have been reported worldwide. Vancomycin-intermediate *S. aureus* (VISA) was first reported in Japan in 1996, and vancomycin-resistant *S. aureus* (VRSA), with a vancomycin minimal inhibitory concentration (MIC) exceeding 128 µg/mL, was first isolated in USA in 2002 [3]. Since the first reported VISA case in 1998 in South Korea, VISA strains have been continuously isolated and are being detected more frequently. However, no cases of VRSA have been identified in South Korea at present [4].

Studies on *S. aureus* with reduced vancomycin susceptibility (SA-RVS; defined as MIC ≥ 1.5 µg/mL or >1.0 µg/mL) and *S. aureus* strains showing a vancomycin MIC in the susceptibility range (≤2 µg/mL) but with increased MIC are gaining interest. Several studies on SA-RVS strains have been published; systematic reviews and meta-analyses on these papers have also been published [5,6,7,8,9,10,11,12]. Although the results are inconsistent to an extent among these studies, several studies similarly report high mortality and treatment failure rates and poor prognosis in infections caused by strains showing increased vancomycin MIC [5,6,7,8,9]. Thus, resistance to vancomycin treatment may be observed even in vancomycin-susceptible strains if the MIC is high, especially at 2 µg/mL, which is the upper limit of the susceptibility range. Consequently, determining the correct MIC values and not just the interpretive category (susceptible, intermediate, and resistant) is important for treatment.

Vancomycin MICs may vary depending on the method of antimicrobial susceptibility test used [13,14,15]. Commercialized automation systems vary depending on the manufacturer, and although the basic principle follows a standard method, and the method was validated, the method and results are not entirely consistent. In this study, we evaluated different antimicrobial susceptibility testing methods to determine vancomycin susceptibility and MIC for SA-RVS isolated from a clinical microbiology laboratory.

## 2. Materials and Methods

In total, 128 clinical isolates of *S. aureus* were tested. To include as many SA-RVS isolates as possible, 99 *S. aureus* isolates were included that showed an MIC of ≥2 µg/mL using the VITEK2 system. Bacterial identification was performed using matrix-assisted laser desorption/ionization mass spectrometry (VITEK MS, bioMérieux, Marcy-l’Étoile, France).

Antimicrobial susceptibility tests were performed using the VITEK2 system with a P601 panel (VITEK2; bioMérieux), Sensititre system with a GPALL1F plate (Sensititre; Thermo Scientific, Waltham, MA, USA), Phoenix M50 system with a PMIC84 panel (Phoenix; BD Diagnostic Systems, Franklin Lakes, NJ, USA), and MicroScan WalkAway 96 Plus system with a Pos MIC 28 panel (MicroScan; Beckman Coulter, Inc., Brea, CA, USA). The Etest (bioMérieux) was performed according to the manufacturer’s instructions, and Etest MICs between standard dilutions were rounded up to the nearest 2-fold dilution. Broth microdilution (BMD) was performed by using 96-well broth microdilution panels according to Clinical and Laboratory Standards Institute (CLSI) guidelines. All antimicrobial susceptibility tests were performed following quality control according to the manufacturer’s instructions or CLSI guidelines. Antimicrobial susceptibility test results of vancomycin were interpreted according to the CLSI guidelines and categorized with certain modifications as follows: ≤1 μg/mL indicated fully susceptible, >1 and ≤2 μg/mL indicated RVS, >2 and <16 μg/mL indicated intermediate susceptibility, and ≥16 μg/mL indicated resistance.

The results of the different antimicrobial susceptibility testing methods were compared with those of BMD as the reference method. Sensitivity, specificity, and positive and negative predictive values (PPV and NPV, respectively) of each method for the detection of SA-RVS were calculated to evaluate performance. Agreement was assessed based on comparison of category (qualitative) and MIC (quantitative). Category agreement was defined as a case in which the results of the interpretive category (susceptible, intermediate, and resistant) were in agreement. A minor error was considered when an isolate was categorized as intermediate via one test method but either susceptible or resistant via another test method. If the MIC determined in the test method was within a single 2-fold dilution (±1 doubling dilution) of the reference result, then the MIC for that isolate was defined as being in agreement (essential agreement).

## 3. Results

Vancomycin MIC determined via BMD ranged from 0.5–2 µg/mL, and all isolates were susceptible to vancomycin; 109 isolates were fully susceptible, and 19 isolates showed RVS. VISA and VRSA strains were not identified. The distributions of the MICs determined via BMD and Etest are shown in Table 1. The BMD and Etest showed complete essential agreement (100%). When BMD results were compared with Etest results, the interpretive category results matched for 120 isolates (category agreement, 93.8%). Minor errors were observed in eight isolates, all of which showed an MIC of 3 μg/mL via Etest. The MIC determined via Etest tended to be higher than that determined via BMD; 71.9% of the Etest results showed a higher MIC than that in the BMD results.

The testing methods were compared with BMD as the reference method (Table 2). Sensititre and Phoenix test results showed complete essential agreement (100%), and VITEK2 showed a high essential agreement (99.2%). MicroScan results showed the lowest essential agreement of 92.2%. VITEK2, Sensititre, and Phoenix test results showed category agreements of 96.1%, 96.1%, and 99.2%, respectively, while MicroScan results showed the lowest category agreement of 85.2%. For all minor errors, the testing method results showed intermediate susceptibility and BMD results showed susceptibility.

The distributions of MICs determined via BMD and testing methods are shown in Table 3. The MIC determined via VITEK2 and MicroScan tended to be higher than that determined via BMD; the proportion of results showing higher MIC than that determined via BMD was 69.5% and 87.5% for VITEK and MicroScan, respectively. In contrast, 82.0% and 84.4% of the MIC results determined via Sensititre and Phoenix were in agreement with BMD, respectively.

The proportions of SA-RVS (%) as determined via Etest, VITEK2, Sensititre, Phoenix, and MicroScan were 68.8%, 73.4%, 15.6%, 20.3%, and 75.0%, respectively. The performance of different antimicrobial susceptibility testing methods for the detection of SA-RVS is shown in Table 4. Phoenix showed high sensitivity and specificity of 84.2% and 90.8%, respectively. The PPV of Sensititre and Phoenix was approximately 60%, and the NPV was >90%. Etest and VITEK2 showed low PPVs of 12.5% and 13.8%, respectively. MicroScan showed the lowest PPV of 4.2%.

When combined with Sensititre or Phoenix, the PPV of Etest and VITEK2 was significantly increased to more than 50%, but the PPV of MicroScan was less than 30%. When Sensititre and Phoenix were combined, PPV was not significantly higher than when each alone (Table 5).

## 4. Discussion

In this study, vancomycin MIC results differed depending on the test used. Although the essential agreement rate with BMD results was high, the MIC values did not match completely in many cases; the essential agreement rates of Etest and VITEK2 were 100% and 99.2%, respectively, while the MICs determined via Etest and VITEK2 were higher than those determined via BMD in 71.9% and 69.5% cases, respectively. Therefore, the vancomycin MICs determined via Etest and VITEK2 tended to be higher than those determined via BMD. A significant proportion of SA-RVS isolates (81/94) initially identified via VITEK2 were found to be vancomycin susceptible when examined using other testing methods. MicroScan showed the lowest essential agreement and category agreement. The MICs determined via MicroScan were higher than those determined via BMD in 87.5% cases and matched completely in 11.7% cases. Additionally, 19 isolates were incorrectly identified as VISA strains using MicroScan. The PPVs for SA-RVS identified using Etest, VITEK2, and MicroScan were considerably low (12.5%, 13.8%, and 4.2%, respectively); therefore, RVS results of these methods should be re-evaluated using additional confirmation tests. In particular, when Etest and VITEK 2 are combined with Sensititre or Phoenix, PPV is significantly improved, so it will be useful if used as a confirmation test. Since many SA-RVS isolates were included in this study to evaluate different antimicrobial susceptibility testing methods to determine vancomycin susceptibility and MIC for SA-RVS isolates, the proportion of SA-RVS isolates was significantly higher than that reported in the normal clinical setting, and the prevalence is lower in the actual clinical setting. Since the PPV reduces as the prevalence is lowered, the low PPV of the testing methods represents a problem. However, in this study, Sensititre and Phoenix showed relatively reliable results for the detection of SA-RVS. The differences between these automation systems may be due to differences in the measurement methods of MICs and experimental conditions according to the testing methods.

Antimicrobial susceptibility tests performed in clinical microbiology laboratories are primarily performed using commercialized automation systems as part of antimicrobial susceptibility test panels that contain various drugs. A previously published study examined and compared vancomycin MICs using the BMD, Etest, and three automation systems, including VITEK2, MicroScan, and Phoenix [16]. They founded that the absolute agreement (0 ± dilution) compared to the BMD was highest for the Phoenix system (66.2%) and the MicroScan turbidity method (61.8%), followed by the VITEK2 system (54.3%). In addition, the Etest produced MIC values one to two dilutions higher than those produced by the BMD method (36.7% agreement). Of interest, the MicroScan system was more likely to overcall an MIC value. In another study, vancomycin MICs for MRSA isolates were determined by agar dilution and the Etest and using the MicroScan, VITEK2, and Phoenix automated systems [17]. They founded that the proportions of MRSA isolate as having high (≥2 mg/L) vancomycin varied depending on the method; the proportions as determined via the agar dilution, Etest, MicroScan, VITEK2, and Phoenix methods were 14.2%, 9.7%, 28.8%, 22.6%, and 3.1%, respectively. In addition, high vancomycin MICs (≥2 mg/L) determined using all three automated systems failed to predict mortality. Therefore, the MIC results obtained using automation systems in clinical microbiology laboratories should be confirmed when necessary, especially when the MIC is high. According to a recent recommendation by the International Working Group, if the vancomycin MIC exceeds 1 µg/mL, a retest or confirmation via other methods is required [18].

This study had a few limitations. The discrepancies observed in this study may be partially attributed to the selection of strains, which favored SA-RVS strains and included more isolates for which vancomycin MICs are close to the intermediate breakpoint than that reported in the actual clinical setting. In this study, we not only reviewed the agreement of each method with the reference method but also focused on and analyzed differences in MICs. However, it is usually considered that an MIC within a single two-fold dilution (±1 doubling dilution) is an acceptable error. Based on this perspective, it may be inappropriate to assume a difference in MICs between the testing methods and reference methods. However, a single two-fold dilution (±1 doubling dilution) difference in MIC can be important in certain cases, especially at a concentration close to the breakpoints for interpretive category determination. There is still a lack of comparative evaluation data between each antimicrobial susceptibility testing method for vancomycin susceptibility in *S. aureus*, especially regarding testing method recently introduced, and it is difficult to generalize the results of this study. This study provides useful information in that it focuses on SA-RVS and evaluated different antimicrobial susceptibility testing methods to determine vancomycin susceptibility and MIC for SA-RVS, including the recently introduced testing method, the Sensititre system. Further studies are needed to determine the most appropriate method for vancomycin susceptibility testing of *S. aureus* in clinical microbiology laboratories. In particular, extensive evaluation is necessary at a high MIC range (>1 µg/mL).

In conclusion, caution should be taken when interpreting RVS and intermediate results for *S. aureus* using the automation system and Etest because strains are likely to be classified as susceptible when tested using other antimicrobial susceptibility tests. In particular, the confirmation of testing results using other methods is strongly encouraged for intermediate resistance results. For confirmation of SA-RVS results, it would be appropriate to test with BMD or a more reliable testing method (Sensititre and Phoenix in this study). An accurate vancomycin MIC may be required for the optimization of antimicrobial dosing. This will provide valuable information on vancomycin susceptibility testing for *S. aureus* and contribute to establishing a reliable and reasonable test method for SA-RVS detection and accurate vancomycin MIC measurement.

## Figures and Tables

**Table 1:** Distribution of vancomycin MICs determined by Etest compared to broth microdilution (BMD).

MIC (μg/mL) Determined by Etest	MIC (μg/mL) Determined by BMD			Sum
	0.5	1	2	
0.5		1		1
0.75	4	3		7
1	3	21		34
1.5		54	2	56
2		23	9	32
3			8	8
Sum	7	102	19	128

**Table 2:** Agreements of vancomycin susceptibility results between different antimicrobial susceptibility testing methods and broth microdilution (BMD).

Testing Method	Agreement with BMD (%)				No. of Minor Error
	Essential Agreement		Category Agreement		
VITEK2	99.2	(127/128)	96.1	(123/128)	5
Sensititre	100.0	(128/128)	96.1	(123/128)	5
Phoenix	100.0	(128/128)	99.2	(127/128)	1
MicroScan	92.2	(118/128)	85.2	(109/128)	19

**Table 3:** Distribution of vancomycin MICs determined by different antimicrobial susceptibility testing methods compared to broth microdilution (BMD).

Testing Method	MIC (μg/mL)	MIC (μg/mL) Determined by BMD			Sum	Testing Method	MIC (μg/mL)	MIC (μg/mL) Determined by BMD			Sum
		0.5	1	2				0.5	1	2	
VITEK2	0.5	3	4		7	Phoenix	0.5				0
	1	3	18	1	22		1	7	92	2	101
	2	1	80	13	94		2		10	16	26
	4			5	5		4			1	1
Sensititre	0.5		1		1	MicroScan	0.5		1		1
	1	7	93	2	102		1	1	11		12
	2		8	12	20		2	6	86	4	96
	4			5	5		4		4	15	19
	Sum	7	102	19	128		Sum	7	102	19	128

**Table 4:** The performance for detection of reduced vancomycin susceptibility in *S. aureus* of different antimicrobial susceptibility testing methods.

Testing Method		BMD (No. of Isolates)			Sensitivity (%)	Specificity (%)	PPV (%)	NPV (%)
		FS	RVS	I				
Etest	FS	32	0	0	57.9	29.4	12.5	80.0
	RVS	77	11	0				
	I	0	8	0				
VITEK2	FS	28	1	0	68.4	25.7	13.8	82.4
	RVS	81	13	0				
	I	0	5	0				
Sensititre	FS	101	2	0	63.2	92.7	60.0	93.5
	RVS	8	12	0				
	I	0	5	0				
Phoenix	FS	99	2	0	84.2	90.8	61.5	97.1
	RVS	10	16	0				
	I	0	1	0				
MicroScan	FS	13	0	0	21.1	15.6	4.2	53.1
	RVS	92	4	0				
	I	4	15	0				

Abbreviations: BMD, broth microdilution; FS, fully susceptible; RVS, reduced vancomycin susceptibility; I, intermediate; PPV, positive predictive value; NPV, negative predictive value.

**Table 5:** Positive predictive value (PPV) for detecting SA-RVS of antimicrobial susceptibility testing methods when each test method and test method are combined.

SA-RVS Results from	PPV (%)	*p*-Value
Etest		
Etest only	12.5	
Etest and Sensititre	56.3	0.0002
Etest and Phoenix	50.0	0.0007
VITEK2		
VITEK2 only	13.8	
VITEK2 and Sensititre	57.9	0.0001
VITEK2 and Phoenix	52.4	0.0003
MicroScan		
MicroScan only	4.2	
MicroScan and Sensititre	28.6	0.0687
MicroScan and Phoenix	25.0	0.1029
Sensititre		
Sensititre only	60.0	
Sensititre and Phoenix	64.7	0.9631
Phoenix		
Phoenix only	61.5	
Phoenix and Sensititre	64.7	0.9121

Abbreviations: SA-RVS, *S. aureus* with reduced vancomycin susceptibility.

## Data Availability

Data of this study can be available on request.
